# Editorial: Inequities and disparities in reproductive health: HIV and STIs

**DOI:** 10.3389/frph.2025.1595001

**Published:** 2025-04-25

**Authors:** Mat Lowe, Choolwe Jacobs, Ruth Oladele

**Affiliations:** ^1^ Society for the Study of Women’s Health (SSWH), Kanifing, Gambia; ^2^ Department for Epidemiology and Biostatistics, University of Zambia, Lusaka, Zambia; ^3^ Department of Physiotherapy, College of Medicine, University of Ibadan, Ibadan, Nigeria; ^4^ Slum and Rural Health Initiative, Ibadan, Nigeria

**Keywords:** inequities and inequalities in health, reproductive health, disparities, STIs - sexually transmitted infections, HIV - human immunodeficiency virus, contraceptive

**Editorial on the Research Topic**
Inequities and disparities in reproductive health: HIV and STIs

Reproductive health affects everyone; however, there are still massive disparities in outcomes and access to care. This editorial contains contributions that bring together research findings on some of the most important inequity issues in the field of reproductive health, including gender, HIV, drug inequity, intervention mapping, determinants of contraceptive use, and associated factors and predictors of loss to follow-up in HIV programs in different country contexts.

A total of thirteen (13) submissions were received for this research topic, with nine accepted and four rejected. The published studies encompass both original research and articles involving secondary data analysis, along with one perspective paper. Three studies from Ethiopia focused on risky sexual practices and associated factors among individuals on antiretroviral therapy (ART) and also examined geospatial variations and determinants of contraceptive utilization among married women of reproductive age, together with the incidence and predictors of loss to follow-up among pregnant and lactating women in the Option B+ Prevention of Mother-to-Child Transmission (PMTCT) program.

The study by Salato et al. among 398 adults living with HIV in South Ethiopia who were undergoing ART revealed a high prevalence (43.7%) of high-risk sexual practices in the six months before the study took place. The results indicated that non-disclosure of HIV status, alcohol consumption, and poor social support were significantly associated with these risky behaviors. The study by Terefe et al. aimed to identify geospatial variations in and determinants of contraceptive utilization among married women of reproductive age in Ethiopia, revealing comparatively low contraceptive usage with considerable regional variation. The study by Azanaw et al. on the incidence and predictors of loss to follow-up among pregnant and lactating women in the Option B+ PMTCT program showed a low incidence of loss to follow-up, which was lower than that reported in the majority of African countries but slightly higher than the World Health Organization (WHO) target.

Gedefie et al. utilized multi-level analysis to assess the prevalence and determinants of HIV among women of reproductive age (15–49 years) in Africa from 2010 to 2019. This study discovered that the highest percentage of HIV was found in Lesotho (23.98%), followed by South Africa (19.12%) and Mozambique (14.67%), emphasizing the need for targeted public health intervention strategies to prevent HIV transmission in these groups.

A systematic review by Zhan et al. examined the prevalence of mental conditions among young people living with HIV (YLWH) and found a heightened risk of mental health issues (24.6% of the participants had depression and 17.0% had anxiety) in this population, underscoring the necessity for targeted interventions to promote the mental health and well-being of YLWH.

Other contributions to this research topic included a qualitative study to understand inequalities in the provision of adolescent sexual and reproductive health services in selected regions of Zambia. The article by Munakampe et al. found that while Zambian adolescents were aware of and had access to common services and commodities such as male condoms, health education, and HIV counseling and testing, availability was hampered by access-related barriers such as frequent stock-outs and insufficiently trained healthcare providers. Accessibility was further reduced during the COVID-19 pandemic lockdown, compounded by low accessibility of sexual and reproductive health (SRH) services among adolescents, which led to the use of alternatives such as herbal medicine and the perpetuation of myths and misconceptions.

Another article by Torres-Cortés et al. sought to promote equity in adolescent health in Latin America through an exploratory sequential mixed-methods study that included literature reviews, focus groups, individual interviews, and intervention mapping to inform the design of a comprehensive sex education program. The authors showed a statistically significant increase in protective skills related to sexuality among all participants following the intervention. Finally, two contributions from the United States (Chory and Bond; Hammond et al.) discussed access to PrEP (pre-exposure prophylaxis) and other sexual health services for cisgender women and the use of PEPFAR (the U.S. President's Emergency Plan for AIDS Relief) to promote equity in access to HIV medications.

## Conclusion

The papers in this research topic highlight significant differences in contraceptive use and disparities in access to HIV and STI prevention and treatment in Ethiopia, Zambia, Latin America, and the U.S., and call for the urgent need for targeted interventions, policy reforms, and greater community engagement.

